# Sirtuin 7 Deficiency Reduces Inflammation and Tubular Damage Induced by an Episode of Acute Kidney Injury

**DOI:** 10.3390/ijms23052573

**Published:** 2022-02-25

**Authors:** Andrea Sánchez-Navarro, Miguel Ángel Martínez-Rojas, Adrián Albarrán-Godinez, Rosalba Pérez-Villalva, Johan Auwerx, Abigail de la Cruz, Lilia G. Noriega, Florencia Rosetti, Norma A. Bobadilla

**Affiliations:** 1 Molecular Physiology Unit, Instituto de Investigaciones Biomedicas, Universidad Nacional Autónoma de México, Mexico City 14080, Mexico; andrea_26sn@hotmail.com (A.S.-N.); mikemarm_93@live.com.mx (M.Á.M.-R.); adrianbiomed@gmail.com (A.A.-G.); melibiosa@hotmail.com (R.P.-V.); 2 Departments of Nephrology and Mineral Metabolism, Instituto Nacional de Ciencias Médicas y Nutrición, Salvador Zubirán, Mexico City 14080, Mexico; 3 Laboratory of Integrative Systems Physiology (LISP), Ecole Polytechnique Federale de Lausanne, CH-1015 Lausanne, Switzerland; admin.auwerx@epfl.ch; 4 Immunology and Rheumatology, Instituto Nacional de Ciencias Médicas y Nutrición, Salvador Zubirán, Mexico City 14080, Mexico; fanby13@gmail.com (A.d.l.C.); florencia.rosettis@incmnsz.mx (F.R.); 5 Nutrition Physiology, Instituto Nacional de Ciencias Médicas y Nutrición, Salvador Zubirán, Mexico City 14080, Mexico; lgnoriegal@gmail.com

**Keywords:** histone deacetylase, NFkB signaling, immune cells infiltration, tubular injury

## Abstract

Acute kidney injury (AKI) is a public health problem worldwide. Sirtuins are a family of seven NAD+-dependent deacylases, Overexpression of Sirtuin 1, 3, and 5 protect against AKI. However, the role of Sirtuin 7 (Sirt7) in AKI is not known. Here, we analyzed how Sirt7 deficient mice (KO-Sirt7) were affected by AKI. As expected, wild-type and Sirt7 heterozygotes mice that underwent renal ischemia/reperfusion (IR) exhibited the characteristic hallmarks of AKI: renal dysfunction, tubular damage, albuminuria, increased oxidative stress, and renal inflammation. In contrast, the KO-Sirt7+IR mice were protected from AKI, exhibiting lesser albuminuria and reduction in urinary biomarkers of tubular damage, despite similar renal dysfunction. The renoprotection in the Sirt7-KO+IR group was associated with reduced kidney weight, minor expression of inflammatory cytokines and less renal infiltration of inflammatory cells. This anti-inflammatory effect was related to diminished p65 expression and in its active phosphorylation, as well as by a reduction in p65 nuclear translocation. Sirt7 deficient mice are protected from AKI, suggesting that this histone deacetylase promotes tubular damage and renal inflammation. Therefore, our findings indicate that Sirt7 inhibitors may be an attractive therapeutic target to reduce NFκB signaling.

## 1. Introduction

Acute kidney injury (AKI) affects 21% of hospitalized patients and up to 60% in critical care units [1,2]. Most of the AKI events are related to ischemic processes in which the hypoxic state, together with the resulting oxidative stress, leads to injury of proximal tubular epithelial cells [3,4,5]. These processes are also accompanied by macrophage infiltration and inflammation [4,6]. Although the tubular epithelium is regenerated, maladaptive mechanisms may take place, leading to adverse, long-term consequences in renal function and structure [7,8].

In recent years, the relevance of histone deacetylation modifications controlling cellular processes has been established; four types of deacetylases have been described and divided into class I, II, III, and IV. Specifically, class III deacetylases are known as sirtuins. Sirtuins are a family of seven NAD+-dependent deacetylases named from 1 to 7, with Sirtuin 7 (Sirt7) being the least studied so far. Sirtuins have been implicated in the development of various human pathologies, including cancer, type II diabetes, dyslipidemias, cardiovascular diseases, or neurodegenerative disorders. Sirt6 and Sirt7 are canonically found in the nucleus, Sirt3 to 5 are mainly located in the mitochondria, and Sirt1 and 2 are found in the nucleus and cytoplasm [9]. Sirt7 regulates cell homeostasis, as it participates in multiple cellular processes such as: gene transcription, ribosome synthesis, chromatin conformation, and cellular proliferation. Sirt7 regulates gene expression, principally by deacetylating histone 3 at lysine residue 18 (H3K18) [10], which represses gene transcription [11]. Sirt7 has also been reported to function as a vital regulator to mitigate stress conditions by activating cell survival mediators. Consistent with this, in some types of cancer Sirt7 overexpression has been associated with increased proliferation and cell survival, as well as angiotensin II-associated cardiac fibrosis [12]. In contrast, Sirt7 depletion is associated with increased DNA damage, apoptosis, chronic hepatosteatosis, and atherosclerosis [13,14,15,16].

Due to the wide spectrum of functions that Sirt7 can have, it is necessary to study its participation in different pathophysiological scenarios [9]. In this context, several studies have reported that the use of histone deacetylase inhibitors is capable to prevent fibrotic and inflammatory processes [17]. 

Sirtuins are highly expressed in the kidney; therefore, understanding the molecular mechanism through which sirtuins participate in renal physiology and how they are altered during AKI results are relevant. Until now, evidence has shown that activation or overexpression of Sirt1, Sirt3, and Sirt5 protects against AKI [18,19,20]. For example, increasing Sirt1 expression in the kidney’s proximal tubules attenuated cisplatin-induced AKI, preserving peroxisome number and function, maintaining catalase activity, and avoiding ROS production [21]. Furthermore, Sirt3 also protects against AKI by improving mitochondrial function [22], and by modulating the Dynamin-related Protein (DRP1) [23] or the AMPK/mTOR pathway [24] to induce mitochondrial autophagy. Finally, SIRT5 protects against AKI by regulating fatty acid oxidation in proximal tubules [25], preserving mitochondrial function, and regulating NRF2 [26]. 

Although there is little evidence about the physiological role of Sirt7 in kidney physiology, it was recently shown that this sirtuin negatively regulates the expression of HIF-1α and HIF-2α, suggesting that Sirt7 is involved in hypoxia-induced signaling [27]. This suggests that Sirt7 may be implicated in AKI since activation of HIF1α significantly reduces apoptosis, macrophage infiltration, and vascular cell adhesion molecule 1 (VCAM1) during ischemic kidney injury [28], and inhibition of HIF1α with a short interfering RNA exacerbates renal IR injury [29]. Additionally, Sirt7 has been shown to be involved in the regulation of fibrotic and inflammatory processes [30], supporting the hypothesis that Sirt7 may be implicated in AKI. 

Because Sirt7 regulates biological processes such as inflammation, hypoxia, oxidative stress, proliferation, and cell survival, all affected during AKI, this study was designed to analyze the response of Sirt7 deficient mice (KO-Sirt7) in this frequent renal pathology. 

## 2. Results

### 2.1. Sirt7 Deficiency in Kidney Dysfunction

**Figure 1:**
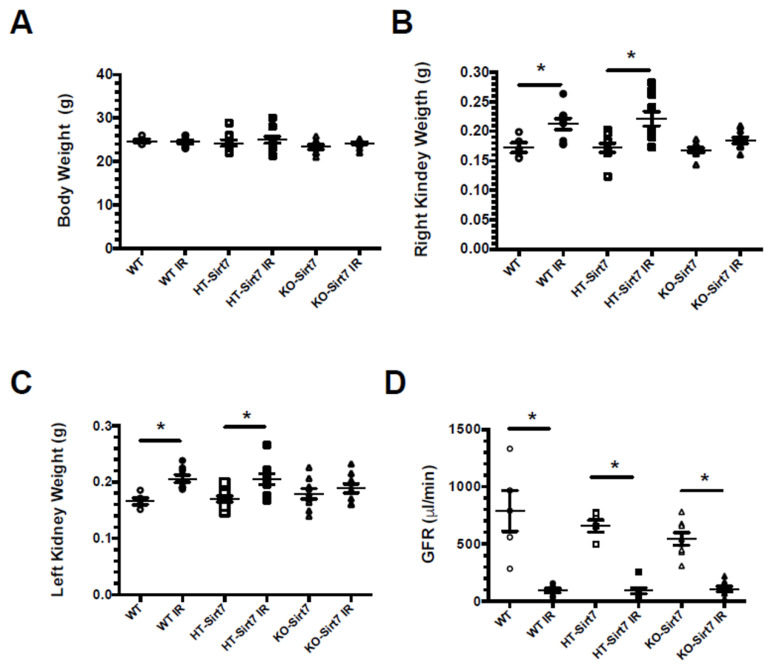
Effect of Sirt7 deficiency on the response to AKI. (**A**) Body weight, (**B**) Right kidney weight, (**C**) Left kidney weight, (**D**) Glomerular filtration rate. White circles represented WT, Black circles, WT+IR, White squares, HT-Sirt7, Black squares, HT-Sirt7+IR, White triangles, KO-Sirt7, and Black triangles, KO-Sirt7+IR. *n* = 5–10 per group. * *p* < 0.05 vs. the respective control group, as stated.

All mice were studied after 24 h of either sham surgery or IR injury. There was no significant difference in body weight among the groups (Figure 1A). Right and left kidney weights were significantly higher in the WT+IR and HT-Sirt7+IR groups while this effect was not observed in the KO-Sirt7+IR group (Figure 1B,C, respectively). This initial finding made us suspect that the KO-Sirt7+IR group exhibited blunted renal inflammation in response to IR.

AKI was also evidenced by the significant reduction in GFR evaluated by the injection of FITC-sinistrin. As is displayed in Figure 1D, all groups that underwent bilateral renal IR showed a robust decrease in renal function, evidencing that Sirt7 deficiency had no impact on renal dysfunction induced by IR. These findings were confirmed by serum creatinine assessment. In all the groups which underwent IR injury, the average serum creatinine significantly increased compared with their respective control group. Thus, in the WT+IR, HT-Sirt7+IR, and KO-Sirt7+IR groups, the serum creatine was: 1.80 ± 0.14, 1.90 ± 0.08, and 1.84 ± 0.14 mg/dL, respectively. The values for WT, HT-Sirt7 and KO-Sirt7 groups were: 0.12 ± 0.01, 0.15 ± 0.04, and 0.12 ± 0.02 mg/dL, respectively.

### 2.2. Sirt7 Deficiency Prevented Tubular Damage

**Figure 2:**
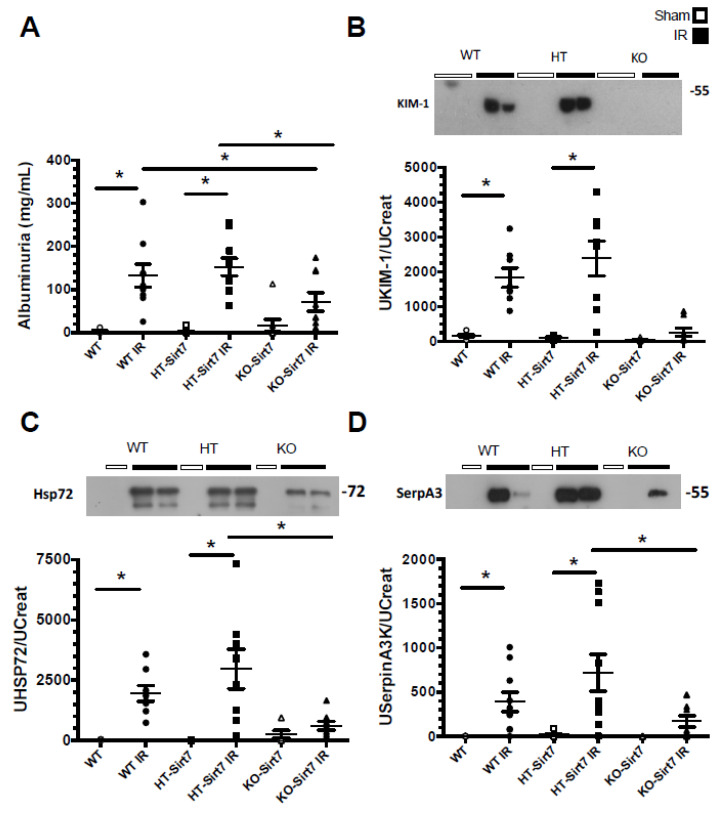
Sirt7 deficiency prevented kidney damage. (**A**) Albuminuria/Urinary Creatinine (UCr) ratio, (**B**) UKIM-1/UCr ratio (**C**) UHSP72/UCr ratio, and (**D**) USerpinA3/UCr ratio. White circles represented WT, Black circles, WT+IR, White squares, HT-Sirt7, Black squares. HT-Sirt7+IR, White triangles. KO-Sirt7, and Black triangles. KO-Sirt7+IR. *n* = 5–10 per group. * *p* < 0.05 vs. the respective control group as stated.

Albuminuria and urinary kidney damage biomarkers were evaluated by ELISA and Western blot, respectively. The renal injury induced by IR was evidenced by a significant increase in albuminuria in WT+IR and HT-Sirt7+IR groups compared to their corresponding sham group. However, albuminuria was attenuated in the KO-Sirt7+IR group (Figure 2A).

The urinary excretion of KIM1, HSP72, and SerpinaA3 biomarkers were evaluated, the last two having been previously described by our group [31,32,33,34,35]. According to our findings, albuminuria, urinary KIM1, HSP72, and SerpinaA3 excretion were significantly increased in the WT+IR and HT-Sirt7+IR groups (Figure 2B–D); however, even though the KO-Sirt7+IR group exhibited renal dysfunction, there was no significant elevation in the biomarkers of kidney injury. 

**Figure 3:**
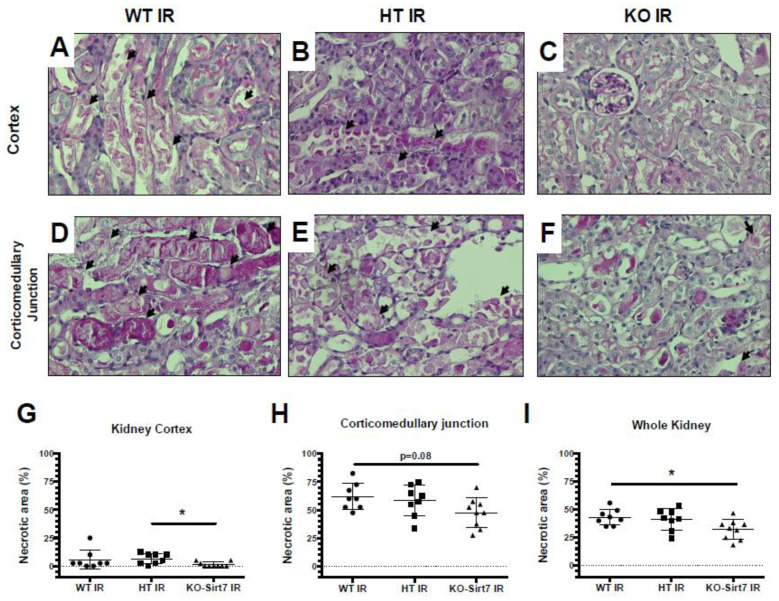
Sirt7 deficiency ameliorated tubular necrosis after IR. (**A**–**F**) Representative PAS images showing necrotic debris (arrows) in kidney cortex (upper panels) and corticomedullary junction (lower panels) of mice subjected to IR. All animals subjected to sham surgery exhibited null necrotic area (not shown). (**G**–**I**) Quantitative analysis of necrotic area in kidney cortex (**G**), corticomedullary junction (**H**), and whole kidney (**I**), indicated lesser tubular injury in KO-Sirt7 animals. * *p* < 0.05 between the indicated groups.

Figure 3 shows representative images of PAS-stained histological kidney sections from the groups studied and their respective blinded quantitative analysis. According to the results of the kidney injury biomarkers, the WT+IR and HT-Sirt7+IR groups exhibited an extensive area of necrosis as can be seen in the photomicrographs depicted in Figure 3A,B,D,E While the tubular damage was lower in the KO-Sirt7+IR group (Figure 3C,F). These findings were confirmed by quantitative analysis of the necrotic area in the renal cortex, corticomedullary junction, and the whole kidney, shown in Figure 3G–I, respectively. These findings suggest that Sirt7 deficiency reduces kidney injury induced by IR.

### 2.3. The Renal Effect of Sirt7 Was Not Mediated by Over-Expression of Other Sirtuins

**Figure 4:**
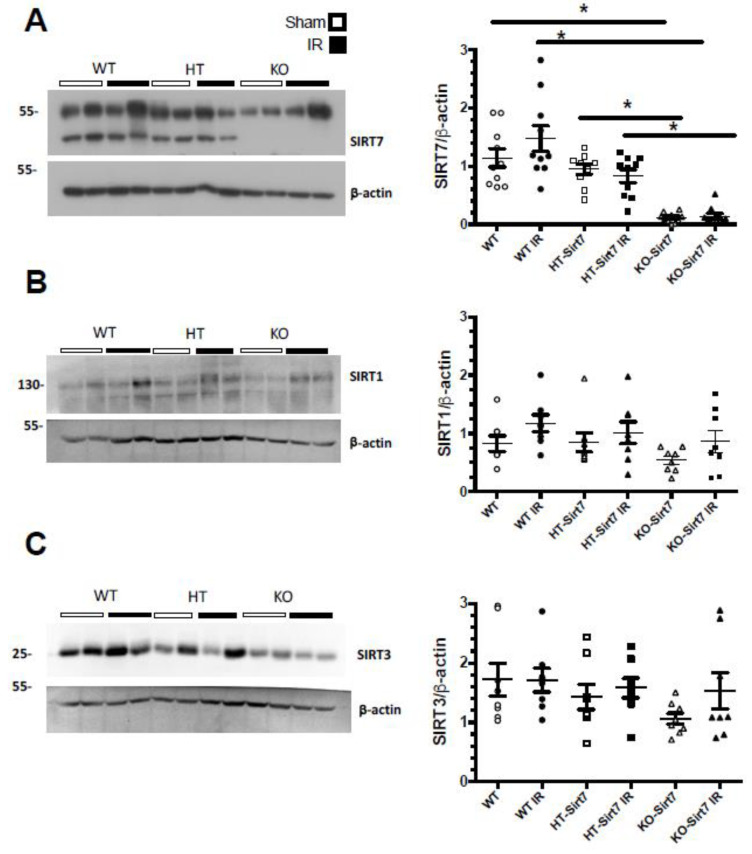
Sirt7, Sirt1 and Sirt3 renal expression. (**A**) Sirt7 protein levels (**B**) Sirt1 protein levels, (**C**) Sirt3 protein levels. White circles represented WT, Black circles, WT+IR, White squares, HT-Sirt7, Black squares, HT-Sirt7+IR, White triangles, KO-Sirt7, and Black triangles, KO-Sirt7+IR. *n* = 5–10 per group. * *p* < 0.05 vs. respective group marked.

The kidney expression of Sirt7 corroborated that KO-Sirt7 mice exhibited a total disappearance of Sirt7 protein levels (Figure 4A). In the case of the WT+IR group, Sirt7 expression tended to increase; however, the difference was not statistically significant (Figure 4A). To study whether Sirt1 and Sirt3 responded to Sirt7 deficiency, we evaluated the protein levels of these two sirtuins. As Figure 4B,D shows, Sirt1 and Sirt3 expression were similar among the studied groups.

### 2.4. Sirt7 Deficiency Reduced Pro-Inflammatory Cytokines’ mRNA Levels

**Figure 5:**
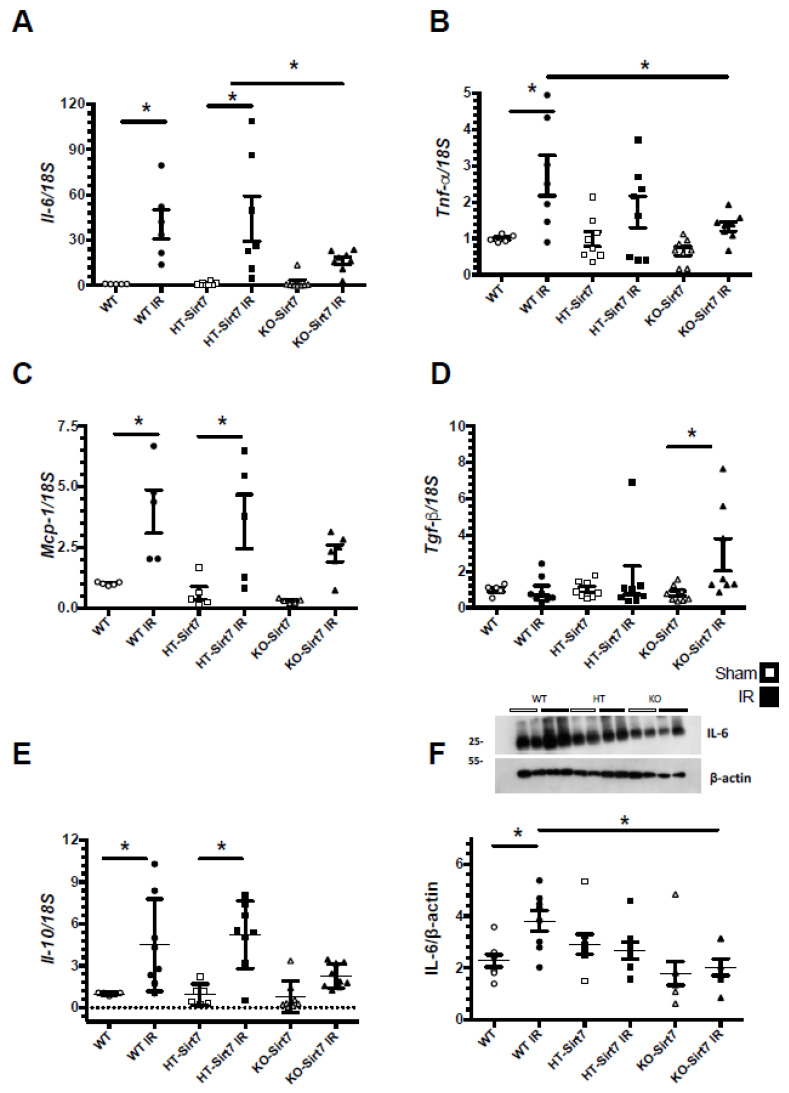
mRNA levels of inflammatory cytokines. (**A**) *Il6*, (**B**) *Tnfa*. (**C**) *Mcp1*, (**D**) *Tgfb*, and (**E**) *Il10* mRNA levels, while in (**F**) are IL-6 protein levels. White circles represented WT, Black circles, WT+IR, White squares, HT-Sirt7, Black squares, HT-Sirt7+IR, White triangles, KO-Sirt7, and Black triangles, KO-Sirt7+IR. *n* = 5–10 per group. * *p* < 0.05 vs. respective group marked.

There was a significant increase in *Il6, Tnfa,* and *Mcp1* mRNA levels in the WT+IR and HT-Sirt7+IR groups compared to their respective control groups: WT and HT (Figure 5A–C). The *Il6* upregulation was corroborated at the protein level (Figure 5F). Interestingly, in the KO-Sirt7+IR group, the expression of pro-inflammatory cytokines was not significantly modified; however, this group exhibited a significant elevation in *Tgfb* mRNA levels, an effect that was not observed in the WT+IR or HT-Sirt7+IR groups (Figure 5D). In addition, there was a significant elevation in *Il10 mRNA levels* in WT+IR and HT-Sirt7+IR groups that was not found in the KO-Sirt7+IR group (Figure 5E).

### 2.5. Sirt7 Deficiency Was Associated with Reduction of Immune Cell Infiltration

**Figure 6:**
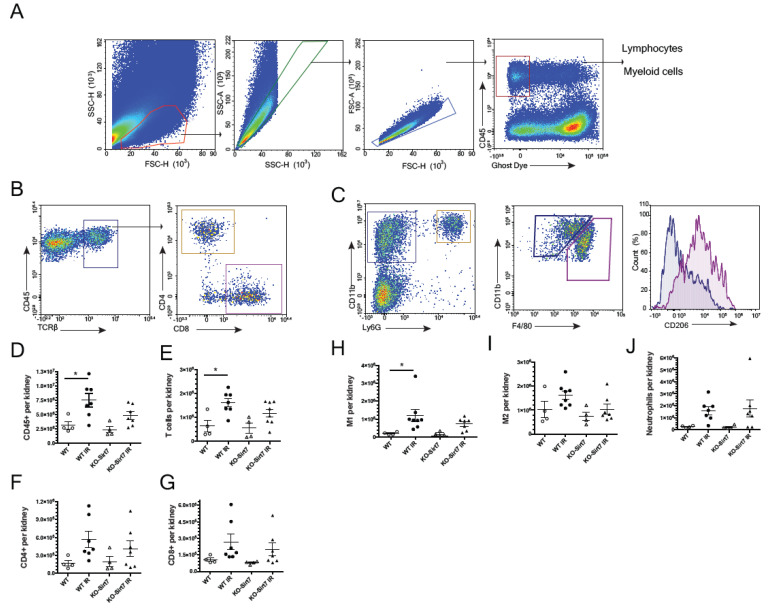
Flow cytometry for T cell and macrophage infiltration. (**A**) Hematopoietic cells (CD45^+^). (**B**) T cells (TCRb^+^), CD4^+^ lymphocytes and CD8^+^ lymphocyte. (**C**) Myeloid cells (CD11b^+^Ly6G^-^). Macrophages M1 (CD11b^+^F4/80l^ow^ and M2 (CD11b^+^F4/80^high^), (**D**) Hematopoietic cells (CD45 +) per kidney, (**E**) T cells (TCRb^+^) per kidney, (**F**) CD4^+^ cells per kidney (**G**) CD8^+^ cells per kidney, (**H**) M1 macrophages per kidney (CD11b^+^F4/80l^ow^), (**I**) M2 macrophages per kidney (CD11b^+^F4/80^high^) and (**J**) Neutrophils per kidney. White circles represented WT, Black circles, WT+IR, White triangles, KO-Sirt7, and Black triangles, KO-Sirt7 IR. *n* = 4–7 per group. * *p* < 0.05 vs. the respective group as stated.

To assess the inflammatory cell infiltrate, we performed a flow cytometric analysis. Figure 6A–C show the methodology used for the gating of the cells using CD45 as a marker of hematopoietic cells. To evaluate T lymphocytes, the presence of T cell receptor beta (TCRb), CD4, and CD8 for T-cell subtypes were analyzed (Figure 6B). For macrophage analysis, M1 and M2 populations were defined based on CD11b and F4/80 expression (CD11b^+^F4/80^low^ and CD11b^+^F4/80^high^, respectively), and CD206 expression was confirmed in the M2 population (Figure 6C). 

Supporting the results on cytokines expression, we found an increased leukocyte infiltration (CD45^+^) upon IR in the WT mice compared to the KO group (*p* = 0.066 vs. WT + IR) (Figure 6D). A significant increase in the total T cell infiltration in the WT+IR group was seen which was not found in the KO + IR group (Figure 6E). No differences in the subpopulation of CD4^+^ and CD8^+^ T cells were found (Figure 6F,G). When macrophage populations were analyzed, we observed an increase in M1 (CD11b^+^F4/80^low^) in the WT group without changes in KO-Sirt7 mice (Figure 6H). No significant changes in M2 (CD11b^+^F4/80^high^) were found amongst the groups (Figure 6I). Neutrophil infiltration seemed not to change among groups (Figure 6J).

### 2.6. Sirt7 Deficiency Reduced the Nuclear Expression of p65

**Figure 7:**
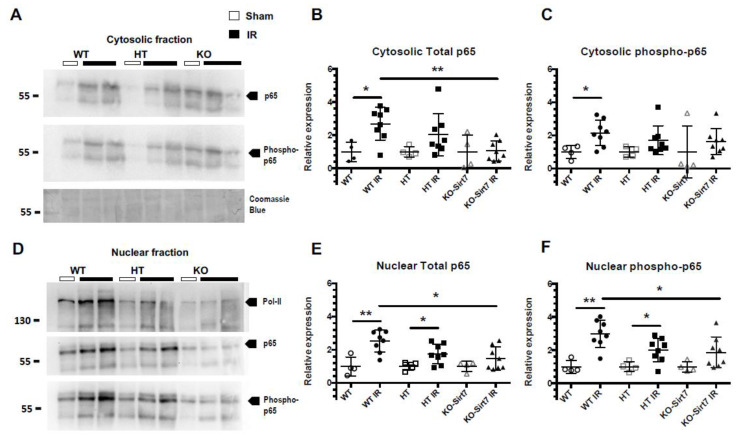
NFkB activation is modulated by SIRT7. (**A**) Protein levels of Total p65, phospho-p65 (Ser 536) and Coomassie Blue in cytosolic fractions. (**B**,**C**) Relative expression of p65 and phospho-p65 in cytosolic fractions based on densitometric analysis. (**D**) Protein levels of polymerase II (Pol II), Total p65, and phospho-p65 (Ser 536) in nuclear fractions. (**E**,**F**) Relative expression of p65 and phospho-p65 in nuclear fractions based on densitometric analysis. White circles represented WT, Black circles, WT+IR, White squares, HT-Sirt7, Black squares, HT-Sirt7+IR, White triangles, KO-Sirt7, and Black triangles, KO-Sirt7+IR. N = nuclei. C = Cytosol. *n* = 5 for control groups and n = 9 for experimental groups. * *p* <0.05 vs. as stated and ** *p* <0.01 vs. as stated.

To evaluate whether Sirt7 modified the NFκB inflammatory pathway, the expression of p65 (a subunit of NFκB) and its phosphorylation (Ser-536) was assessed in renal cytosol and nuclei fractions (Figure 7A,D). Polymerase II expression was used as a control for nuclear extraction. In line with our findings on pro-inflammatory cytokine expression and on cell immune infiltration, the WT+IR and HT-Sirt7+IR groups exhibited a significant increase in the expression of p65 and phospho-p65 in cytosolic fractions as compared with their respective WT and HT groups, an effect that was not seen in the KO-Sirt7+IR group (Figure 7A–C). When p65 translocation to the nuclei was assessed, similar results were found, there was a significant rise in p65 and phospho-p65 in nuclear fractions from WT+IR and HT-Sirt7+IR groups compared to nuclear fractions from WT and HT groups. Interestingly, NFκB signaling activation was reduced in the KO-Sirt7+IR group (Figure 7D–F).

## 3. Discussion

We found that Sirt7 deficiency attenuated AKI, which was evidenced by lower urinary excretion of both albumin and biomarkers of kidney damage, such as Hsp72, KIM-1, and SerpinaA3 [31,32,36]. Although we found less tubular damage in Sirt7 deficient mice, renal dysfunction (GFR) induced by IR was not alleviated, suggesting that this sirtuin has little effect on vasoconstrictor factors that are induced after AKI.

Sirtuins are nicotinamide adenine dinucleotide (NAD+)-dependent lysine deacetylases that regulate various biological processes, such as metabolism, inflammation, genomic stability, stress responses, and aging [37]. One possible explanation for the renoprotection observed after IR in the Sirt7 deficient mice could be mediated by a compensatory overexpression of Sirt1 and Sirt3 that have been previously described as protectors from AKI [17,18,19]. However, this was not the case because Sirt1 and Sirt3 expression were not different between WT and KO mice, indicating that the renal benefits were mediated by Sirt7 deficiency and their targeted pathways.

Another interesting finding of this study was that the KO-Sirt7+IR group did not exhibit an increase in kidney weight induced by ischemic injury, which strongly suggests that Sirt7 deficiency is associated with reduced renal inflammation. Indeed, this group exhibited a lower elevation of pro-inflammatory cytokines such as IL6, TNFα, and MCP-1. Our data is interesting because, despite renal hypoperfusion and hypofiltration in these mice, Sirt7 deficiency mitigated the renal inflammation induced by IR. Supporting our results, Miyasato, Y. et al., reported that Sirt7 deficient mice, suffering from cisplatin nephrotoxicity, exhibited a reduction in *Tnfa, Il1b, Il6, Ccl2*, and *Cxcl2* mRNA levels, as well as in F4/F80 positive cells compared to WT mice [38]. Moreover, it has been reported that Sirt7 deficiency protects against pulmonary endothelium inflammation induced by lipopolysaccharides, an effect that was associated with an increase in TGFβ1 expression and its target genes, driving endothelium-mesenchyme transition to promote cellular repair [39]. Interestingly, we also found that after IR, the KO-Sirt7 mice had a significant increase in *Tgfb1* mRNA levels that was not seen in the WT+IR and HT-Sirt7 groups. Additionally, it has been reported that after an ischemic insult, TGFβ1 not only increases the synthesis of fibronectin and collagen IV, but also regulates the proliferation and differentiation of epithelial tubular cells to facilitate more efficient tubular epithelium restoration [40]. Taking all these findings together, it is very probable that TGFβ1 up-regulation seen in the KO-Sirt7+IR group can help to protect the tubular epithelium injured during AKI. 

Our observation, as well as the findings of Miyasato Y, et al. [38], are somehow different to those observed in aged mice, in which Sirt7 deficiency induced heart inflammation and fibrosis through enhancing TGFβ expression [41,42], or increased the acetylation, and decreased the expression of the K-Cl cotransporter KCC4, leading to an exacerbated metabolic acidosis during an ammonium chloride challenge [43]. These observations suggest that Sirt7 may modulate renal inflammatory state and/or renal physiology in an age-dependent way that could possibly involve an NAD^+^ dependent mechanism. The NAD^+^ pool is decreased during aging [44] and during AKI [45]. The de novo NAD+ biosynthetic pathway is particularly relevant in the kidney to maintain NAD+ levels since it expresses all the NAD+ biosynthetic enzymes. Among them, the α-amino-β-carboxymuconate-ε-semialdehyde decarboxylase (ACMSD) forms α-aminomuconate-ε-semialdehyde (AMS), which can be oxidized to CO_2_ and H_2_O, and, therefore, decreases NAD^+^ synthesis. Notably, pharmacological inhibition of ACMSD increases NAD^+^ levels, improving mitochondrial and kidney function, especially decreasing KIM1 protein content and GFR during AKI induced by cisplatin [45]. Furthermore, the treatment of mice with nicotine riboside, a precursor of NAD+, before the induction of IR, alleviated tubular injury [46]. Nevertheless, further studies are required to understand the implications of NAD and the role of Sirt7 at different stages of life. 

It is important to point out that the reduction in pro-inflammatory cytokines found in the KO-Sirt7 IR group was associated with a small infiltration of CD45 cells and macrophages after 72 h post-ischemic insult, the period during which the M1 to M2 transition takes place [47,48]. NFkB is one of the most studied transcription factors that, after activation, is translocated to the nucleus where it governs a pro-inflammatory response during AKI in many different cells, for instance, it regulates the expression of adhesion molecules such as VECAM in endothelium [49], the expression of cytokines and apoptosis in tubular epithelial cells and monocytes [50], and regulates the population of lymphocytes after renal IR [51]. NFkB is composed of five proteins: NFkB1, NFkB2, RelA (p65), RelB, and c-REL [52]. In this study, we found an increase in the expression and active phosphorylation of p65 in the WT+IR and HT-Sirt7+IR groups. In agreement with the reduced inflammation observed in the Sirt7 deficient mice, nuclear phospho-p65 accumulation was not observed in those mice. Comparable results have been reported in KO-Sirt7 mice with cisplatin nephrotoxicity [38]. Futhermore, Sobuz, S. et al. recently demonstrated that Sirt7 interacts with a small GTPase nuclear antigen related to Ras (Ran), which it deacetylates on the K37 residue, in turn, promoting the nuclear export of NFkB [53]. The effect of Sirt7 deficiency on NFkB is possibly pivotal in renal injury induced by IR, however, we did not evaluate the activity of this transcription factor in a cell-specific manner; therefore, the precise relationship between Sirt7 and NFkB in different cellular subpopulations during AKI requires a deeper exploration.

In summary, this study allowed us to analyze the role of Sirt7 in AKI induced by IR. Our results show that Sirt7 deficiency did not improve renal dysfunction induced by IR, but it has a profound effect in preventing renal inflammation through reducing nuclear phospho-p65 accumulation and, therefore, in minimizing the robust pro-inflammatory activity of NFκB, helping to improve tubular epithelial injury. 

## 4. Material & Methods

All the experimental procedures in the animals were conducted following the Guide for the Care and Use of Laboratory Animals and were approved by the animal research ethics committee at Instituto Nacional de Ciencias Médicas y Nutrición Salvador Zubirán. 

### 4.1. Mouse Model of AKI

We used the Sirt7 deficient mice previously described and mice with a pure C57BL/6 background [54]. Sixty mice at an age of two months were included, 20 of them were wild-type (WT), 20 were heterozygous (HT-Sirt7), and 20 were Sirt7 deficient mice (KO-Sirt7). These mice were divided into six groups: (1) the WT were subjected to sham surgery or (2) underwent bilateral ischemia-reperfusion (IR) for 22.5 min; (3) the HT-Sirt7 mice were subjected to sham surgery and (4) subjected to IR; (5) the KO-Sirt7 mice were subjected to sham surgery, (6) and subjected to IR (KO-Sirt7+IR). Their urine was collected for 18 h, and all the mice were studied for 24 h after surgery. 

### 4.2. Glomerular Filtration Rate Assessment and Tissue Harvesting

The glomerular filtration rate (GFR) was determined by injecting FITC-sinistrin using a fluorescence monitor [55]. The animals were partially sedated with sodium pentobarbital (15 mg/kg) and a fluorescent sensor was placed and fixed on the depilated skin to measure the GFR. A basal reading was made for one minute. Then 7 mg/kg BW of FITC-sinistrin was injected and its decay rate was determined during 1 h to calculate the GFR. At the end of the experiment, blood samples were taken; their kidneys were isolated and divided into two sections. One half was stored at −70 °C for molecular and biochemical analyses, and the other half as fixed for histological analysis.

### 4.3. Histological Evaluation of Tubular Injury

After tissue fixation on 4% paraformaldehyde solution, the kidneys were dehydrated and embedded in paraffin. Then, kidney slices of 4 μm were obtained and stained with periodic acid-Schiff (PAS) to assess tubular integrity. For each mouse kidney, twelve high-power fields (magnification 200×) were captured from kidney cortex and corticomedullary junction using a camera incorporated to the microscope and linked to the NIS-Elements software (Nikon Instruments Inc. Mellville NY, USA) in a blinded fashion. Once digitalized, the necrotic area was quantified and computed for each image. The necrotic debris was identified in tubules with individual or cast-forming cells with increased eosinophilia, partial or total loss of nuclear material (karyolysis, karyorrhexis), and rupture of tubular basement membrane (tubulorrhexis). Finally, data were distributed by kidney section (cortex, corticomedullary junction, whole kidney) in the corresponding groups and compared statistically.

### 4.4. Assessment of Creatinine, Albuminuria, and Urinary Biomarkers of Kidney Damage

Quantichrom ™ creatinine assay kit (DICT-500, Hayward, CA, USA) was used to measure serum creatinine concentration following the manufacturer’s guidelines. Albuminuria was assessed in 10 µL of urine with an ELISA kit (Exocell Hayward, CA, USA), following the manufacturer’s instructions. Urinary biomarkers of kidney damage were evaluated by Western Blot analysis using 0.1 µL of urine diluted in a 0.9% saline solution. Proteins were blotted onto a PVDF membrane. The membranes were blocked with a 5% blocking agent; subsequently, they were incubated overnight at 4 °C with anti-HSP72 (hybridoma, 1:10,000, Mexico City, Mexio), anti-KIM-1 (1:5000, Boster, Cat. No. PA1632, Pleasanton, CA, USA), or with anti-SerpinA3K (Proteintech, Cat. No. 55480-1-AP, 1:5000, Rosemont, IL 60018, USA). A secondary anti-mouse antibody (Santa Cruz, Cat. No. sc-2031, 1:10,000 Dallas, TX, USA) was used for HSP72, or anti-rabbit (Santa Cruz, Cat. No. sc-2004, 1:5000, Dallas, TX, USA) for KIM-1 and serpinA3K. Proteins were detected using a commercial chemiluminescence kit (Millipore, Cat. No. WBKLS0500, Burlington, MA, USA) and normalized by urinary creatinine (UCreat).

### 4.5. Evaluation of Pro-Inflammatory Cytokines mRNA Levels

Total RNA was extracted with the trizol method, and retro-transcription was performed to generate the cDNA. The probes that were used to amplify the genes were: *Il6* (Mm00446190_m1), *Tnfalpha* (Mm0443258_m1), *Mcp1* (Rn00580555_m1), *Tgfb1* (Mm03024053_m1), and *Il10* (Mm01288386_m1). The mRNA level of each one was quantified by real-time PCR on QuantStudio 5 (Life Technologies). Eukaryotic 18S rRNA was used as endogenous control (predesigned assay reagent, external run, Rn03928990_g1, Cat. No. 4319413E was used). The relative quantification of each gene expression was performed with the comparative threshold cycle method (Ct) [56].

### 4.6. Nuclear and Cytoplasmic Protein Extraction

The kidney tissue (50 mg) was minced into very small pieces on a cooled glass plate. Then, it was transferred into an ice-cold Dounce tissue homogenizer. Ten volumes of lysis buffer [10 mM HEPES; pH 7.5, 10 mM KCl, 0.1 mM EDTA, 1 mM dithiothreitol (DTT), and 0.5% Nonidet-40] together with the protease inhibitor cocktail were added. The mix was allowed to sit on ice for 15–20 min with intermittent mixing, and the tissue was disaggregated through 5–10 strokes and sat on ice for 2 min. The lysate was centrifuged at 700 g for 5 min to create a nuclei pellet. The supernatant (cytoplasmic fraction) was transferred to a new tube. The pelleted nuclei were washed thrice, resuspended in 5 volumes of nuclear extraction buffer [20 mM HEPES (pH 7.5), 400 mM NaCl, 1 mM EDTA, 1 mM DTT, and protease inhibitor cocktail], and incubated on ice for 30 min. Nuclear extract was collected by centrifugation at 12,000 *g* for 15 min at 4 °C. 

### 4.7. Protein Expression by Western Blot and Antibodies

Renal cortex proteins were homogenized with a lysis buffer containing: 50 mM HEPES pH 7.4, 250 mM NaCl, 5 mM EDTA, 0.1% NP-40, and complete protease inhibitor (Roche, No. Cat. 11697498001). Protein concentration was evaluated by the Lowry Protein Assay (Bio-Rad, Cat No. 5000113 and 5000114, Hercules, CA, USA). Proteins obtained from each animal were electrophoresed using 20 µg of proteins on an 8.5% acrylamide denaturing gel containing SDS. The membranes were incubated overnight at 4 °C with the primary antibody Sirt7 (Santa Cruz, Cat. No. sc365344, 1:1000, Dallas, TX, USA), Sirt3 (Santa Cruz, Cat. No. sc-365175, 1:1000, Dallas, TX, USA), IL-6 (Santa Cruz, No. Cat. Sc57315, 1:1000, Dallas, TX, USA), NFκB p65 total (F-6, Santa Cruz, No. Cat. sc-8008, 1:1000, Dallas, TX, USA), p-NFκB p65 (27.Ser 536, Santa Cruz, No. Cat. sc-136548, 1:1000, Dallas, TX, USA), Pol II (F-12, Santa Cruz, No. Cat. sc-55492, 1:1000, Dallas, TX, USA), and HRP β-actin antibody [AC-15] (Abcam, Cat. No. ab49900, 1:1,000,000, Cambridge, UK). Three 10 min washes were performed with 1x TBS-Tween, and then incubated with the secondary antibody coupled to HRP, anti-rabbit, or anti-mouse IgG (Santa Cruz, Cat. No. sc-2031 or sc-2004, respectively 1:5000, Dallas, TX, USA). Tissue proteins evaluated by Western blot were normalized by detection of β-actin.

### 4.8. Kidney Cells Flow Cytometry

WT or KO-Sirt7 mice were divided into sham surgery or bilateral renal ischemia (22.5 min) groups. The cellular infiltration, particularly macrophages and lymphocytes, were analyzed 72 h post-ischemia because the greatest inflammatory cell infiltration had been reported at this point. [47,48]. The mice were anesthetized with sodium pentobarbital (30 mg/kg,) and their kidneys were perfused with 20 mL of PBS. One kidney was removed and placed in PBS where it was cut into small pieces with scissors. Collagenase 1 mg/mL and DNAse 50 μg/mL were added and incubated for 30 min at 37° with shaking. A 70 μm cell strainer was used to obtain cell suspension from the digested kidneys. Red blood cells were lysed with ACK lysis buffer. Kidney cells were suspended in PBS to perform a cell count and staining for flow cytometry. The following antibodies were purchased from Tonbo (Tonbo biosciences, San Diego, CA, USA): Ghost dye (violet 510 or APC-Cy7), CD45.2 (APC-Cy7 or APC), F4/80 (VF450), CD11b (PECy7), Ly6G (PE), TCRβ (FITC), CD4 (PerCPCy5.5), CD8 (PE) (Tonbo), and CD206 (APC) (BioLegend, San Diego, CA, USA). All these stains were performed in the presence of Fc block (Tonbo). The samples were read on a NovoCyte Flow Cytometer, Volt Technology (Agilent) and the results obtained were analyzed with NovoExpress software (Agilent, Santa Clara, CA, USA).

### 4.9. Statistical Analysis

The variables are presented as mean ± SEM. Groups were compared with one-way ANOVA and the Bonferroni multiple comparisons post hoc test. Statistical significance was defined as a *p*-value < 0.05. Statistical analyses and graphics were performed on GraphPad Prism Version 9 software (San Diego, CA, USA).

## Data Availability

The data that supports the findings of this study are available on request from the corresponding author (NAB).
